# In Vitro Biotechnology for Conservation and Sustainable Use of Plant Genetic Resources

**DOI:** 10.3390/plants13141897

**Published:** 2024-07-10

**Authors:** Waed Tarraf, Anna De Carlo

**Affiliations:** Institute of BioEconomy, National Research Council (CNR/IBE), Sesto Fiorentino, 50019 Florence, Italy; anna.decarlo@ibe.cnr.it

## 1. Introduction

This Special Issue contains novel contributions related to the role and use of in vitro culture in the conservation and sustainable use of plant genetic resources. This area is of increasing interest in the global community to guarantee adequate supplies of food for future generations. All cultivated, wild relatives of cultivated species, traditional cultivars, landraces, and advanced breeding lines of plants are included in plant genetic resources [1]. Indeed, the increase in world population growth has led to significant pressure being exerted on many valuable plant species, which, in turn, has led to the genetic erosion of important germplasms from their natural habitats [2]. However, the loss of biodiversity due to the overexploitation of natural populations, random harvest at different levels from the wild, natural hazards, population growth, and climate change are considered great threats to plant genetic resources [3]. This risk can be overcome by adopting biotechnological techniques, including rapid and large-scale propagation [4] and genetic improvement in plants [5], to achieve sustainable agricultural production and enhance food security. There are two strategies to conserve biodiversity: (i) in situ conservation, where the plant species are conserved where they are found and are maintained in their original location [6] with minimum interventions from humans, and (ii) ex situ conservation, where plant materials are preserved outside their natural habitats [7]. However, for a more effective ex situ conservation program, plant tissue culture has emerged as an interesting approach to maintaining the germplasm in healthy and controlled conditions [8]. Among these ex situ conservation approaches, micropropagation offers a rapid and efficient method for the large-scale production of economically important plants in a short time and limited space [9]. Additionally, in vitro conservation effectively maintains plant germplasm, especially rare and endangered species, and recalcitrant seed and vegetatively propagated species [10].

This Special Issue comprises ten contributions focused on the role of in vitro culture for the propagation, conservation, and sustainable use of plant genetic resources.

## 2. Novel Approaches for the Conservation of Plant Genetic Resources

The International Union for Conservation of Nature (IUCN) estimates the global conservation status of species, and according to the latest updated data, 26,276 plant species out of 66,536 species, where they evaluated 425,035 plants, are classified as threatened and have been put on the red list of threatened species [11]. Therefore, cultivation of these species is highly recommended to limit the overexploitation practices that seriously threaten the sustainable use of these resources. Jain et al. [12] demonstrated the effectiveness of biotechnological methods for germplasm conservation, including in vitro propagation, genetic transformation, DNA banks, and cryopreservation. These techniques allow for preserving pathogen-free material, elite plants, and genetic diversity in the short-, medium-, and long-term.

Compared to conventional propagation methods, synthetic seeds can offer an efficient alternative tool for the propagation and conservation of valuable plant species that are difficult to propagate [13]. Combining synseed technology with micropropagation represents perfect biotechnology that could be convenient in the agriculture sector to improve the production of year-round plants. Many studies have proven the role of plasma treatments in improving seed germination and accelerating the conversion of plantlets into whole plants [14,15]. For example, seed treatment with high-frequency atmospheric-pressure plasma, often called “cold plasma”, can positively affect seed germination and subsequently ensure plant development of conventional seeds without adding chemicals that are harmful to the environment and human health [16]. In this SI, Škoro et al. [17] applied a novel approach to obtain artificially encapsulated *Chrysanthemum* shoot tips before sowing in the soil. They showed the high efficiency of the surface dielectric barrier discharge air cold plasma treatment in directly developing plasma-treated synthetic seeds into entire plantlets. This treatment significantly improved the agronomic seed quality by minimizing contamination and promoting a considerable regrowth and conversion of *Chrysanthemum* synseeds into whole plantlets, either in vitro (under aseptic) or ex vitro (non-aseptic) conditions. 

Many innovations in in vitro culture protocols for the propagation and breeding program of many species were reported in the present SI. For example, the use of meta-Topolin to improve the micropropagation of the *Lagerstroemia speciosa* [18]. This substance could successfully replace several traditional cytokinins [19,20] and possess an advantage for the in vitro propagation of true-to-type plant material appropriate for market needs, conservation strategies, and pharmaceutical purposes. Particularly, obtaining formulations and industrial products derived from micropropagated *L. speciosa* plants characterized by a higher level of corosolic acid (the future anti-diabetic drug).

This Special Issue included the indirect organogenesis protocol of *Origanum dictamnus* by Sarropoulou et al. [21], which was obtained for the first time. Based on the IUCN classification, it has been evaluated as near-threatened; therefore, the in vitro regeneration of this valuable endemic plant is highly recommended to decrease its overharvesting in nature. The authors developed an efficient protocol for the in vitro indirect regeneration testing of the potential of different plant tissues and organs to regenerate shoots or roots. 

Tan et al. [22] created an interspecific hybrid of *Oryza officinalis* and cultivated rice, verifying the fertility of its pollen and embryo sac using cytological analysis. They obtained an optimal protocol to induce polyploidy by producing high-quality callus followed by colchicine treatment at 400 mg·L^−1^ for 2 days. This work can provide a solution for cross-compatibility in *O. officinalis* and may pave the way for further improvement programs in rice.

Benelli et al. [23] underlined the importance of the slow growth storage (SGS) technique as an efficient in vitro approach for the preservation of many fruit species by controlling their growth under in vitro conditions. It is known as “minimal growth storage” due to changes applied to some physical, chemical, or nutritional factors to decrease the growth of plantlets. Also, it is called “cold storage” when low temperatures replace the standard growth conditions. In this review, the effect of many factors on the SGS of shoot cultures from temperate and tropical species has been deeply discussed and supported by published works during the last ten years.

In the context of SGS, Mendler-Drienyovszki and Magyar-Tábori [24] investigated the cold storage of the genetic resources of endangered species from the genus *Sorbus*. They reported for the first time the cold storage ability of in vitro *S. redliana* shoots at 4 °C under dark conditions. After 52 weeks, the stored shoot cultures maintained 100% survival with an efficient multiplication rate when regrown under normal culture conditions. This result allowed for plant materials without at least three subcultures to be maintained compared to the periodical subculture of four weeks, leading to a reduction in costs and saving working time. 

Also, Tirado et al. [25] evaluated the in vitro conservation of Mexican garlic varieties by minimal growth. The results underlined a maximum storage of one year at 5 °C on a Murashige and Skoog medium supplied with a combination of sucrose and sorbitol. Therefore, optimizing a protocol can be applied to other garlic varieties for medium-term storage in germplasm banks.

Cryopreservation as another biotechnological tool is applied to rescue plant genetic resources that are often at risk of loss due to overexploitation, sometimes combined with other biotic and abiotic stresses, leading to a decline in their natural habitats. The review of El Merzougui et al. [26] presented an update on the application of cryopreservation to medicinal and ornamental geophytes over the last 20 years. Indeed, several factors that limit the success of bulbous germplasm conservation were reported. Such a review will highly help the biologists and cryobiologists in their further research to optimize the cryopreservation protocols of geophytes.

The use of cryopreservation for the preservation of kiwifruit is reported in this SI by Nadarajan et al. [27]. The authors outlined the current status of the kiwifruit collection in New Zealand and detailed the ongoing development of in vitro collection for germplasm conservation. As a result of the spread of *Pseudomonas syringae* pv. *Actinadiae*-biovar 3 in 2010, which had a destructive effect on the health of field collections, almost all the *Actinidia* accessions maintained in field collections are introduced under in vitro conditions. These collections hold about 450 genotypes from many species, so managing such a huge collection requires appropriate protocols. The authors discussed the methods applied for the medium-term storage and long-term conservation of accessions of *Actinidia.*

Moreover, this Special Issue presented unique research on the long-term conservation of conifers that are under immediate risk of extinction. Benelli et al. [28] developed and validated cryopreservation protocols for pollen, zygotic embryos, and embryogenic callus to establish a cryobank for *Abies nebrodensis* Thereby, the authors suggested a strategy that allows for the safe preservation of the remaining population of *A. nebrodensis* and further opens the door for similar initiatives for other critically endangered conifers.

## 3. Conclusions and Future Perspectives

This Special Issue highlighted ex situ conservation as a highly efficient strategy for germplasm preservation. Many novel approaches are discussed, such as synseed technology and cold plasma treatment, the use of meta-Topolin in multiplication medium to replace many traditional cytokinins, and indirect organogenesis of near-threatened species. Also, the Special Issue included different research on in vitro culture technology, in vitro preservation, and cryopreservation. Further efforts are required to promote the in vitro conservation of plant germplasm collection and extend the applications of obtained protocols to other plant species, in particular rare and under-threat species, to ensure the sustainable use of the current plant genetic resources for the next generations. 

## Data Availability

Not applicable.
